# Packaging and Delivery of Chemical Weapons: A *Defensive Trojan Horse* Stratagem in Chromodorid Nudibranchs

**DOI:** 10.1371/journal.pone.0062075

**Published:** 2013-04-19

**Authors:** Marianna Carbone, Margherita Gavagnin, Markus Haber, Yue-Wei Guo, Angelo Fontana, Emiliano Manzo, Gregory Genta-Jouve, Maria Tsoukatou, William B. Rudman, Guido Cimino, Michael T. Ghiselin, Ernesto Mollo

**Affiliations:** 1 Istituto di Chimica Biomolecolare, Consiglio Nazionale delle Ricerche, Pozzuoli, Italy; 2 Department of Zoology, Tel Aviv University, Tel Aviv, Israel; 3 State Key Laboratory of Drug Research, Shanghai Institute of Materia Medica, Chinese Academy of Sciences, Shanghai, China; 4 Institut de Chimie de Nice, Université de Nice-Sophia Antipolis, Nice, France; 5 Department of Pharmacognosy, University of Athens, Athens, Greece; 6 Malacology Department, Australian Museum, Sydney, New South Wales, Australia; 7 Department of Invertebrate Zoology, California Academy of Sciences, San Francisco, California, United States of America; National Cancer Institute at Frederick, United States of America

## Abstract

**Background:**

Storage of secondary metabolites with a putative defensive role occurs in the so-called mantle dermal formations (MDFs) that are located in the more exposed parts of the body of most and very likely all members of an entire family of marine mollusks, the chromodorid nudibranchs (Gastropoda: Opisthobranchia). Given that these structures usually lack a duct system, the mechanism for exudation of their contents remains unclear, as does their adaptive significance. One possible explanation could be that they are adapted so as to be preferentially attacked by predators. The nudibranchs might offer packages containing highly repugnant chemicals along with parts of their bodies to the predators, as a defensive variant of the strategic theme of the Trojan horse.

**Methodology and Principal Findings:**

We detected, by quantitative ^1^H-NMR, extremely high local concentrations of secondary metabolites in the MDFs of six species belonging to five chromodorid genera. The compounds were purified by chromatographic methods and subsequently evaluated for their feeding deterrent properties, obtaining dose-response curves. We found that only distasteful compounds are accumulated in the reservoirs at concentrations that far exceed the values corresponding to maximum deterrent activity in the feeding assays. Other basic evidence, both field and experimental, has been acquired to elucidate the kind of damage that the predators can produce on both the nudibranchs' mantles and the MDFs.

**Significance:**

As a result of a long evolutionary process that has progressively led to the accumulation of defensive chemical weapons in localized anatomical structures, the extant chromodorid nudibranchs remain in place when molested, retracting respiratory and chemosensory organs, but offering readily accessible parts of their body to predators. When these parts are masticated or wounded by predators, breakage of the MDFs results in the release of distasteful compounds at extremely high concentration in a way that maximizes their repugnant impact.

## Introduction

**Figure 1:**
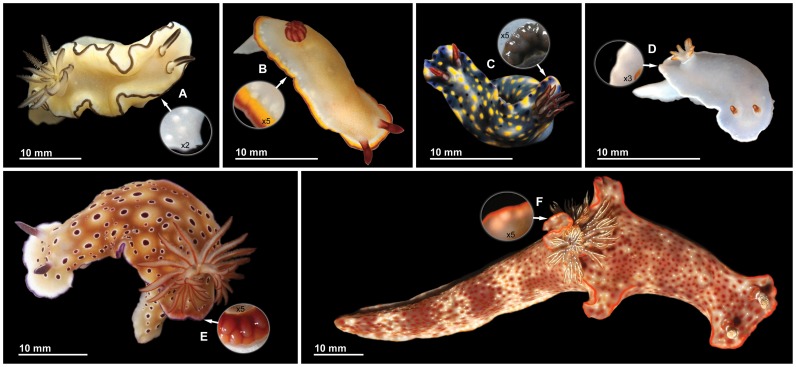
Animals studied. (A) *Glossodoris atromarginata*; (B) *Chromodoris sinensis*; (C) *Hypselodoris infucata*; (D) *Hypselodoris* sp.; (E) *Risbecia tryoni*; (F) *Ceratosoma gracillimum.* Circles indicate mantle regions with MDFs (after dissection from below in C and E).

Chemical ecology has made contributions of fundamental importance to the understanding of molecularly mediated biological interactions and evolutionary patterns [1], [2]. Compounds that mediate biological functions have played an important role in the adaptive radiation and ecological expansion of such dominant terrestrial organisms as insects, and the same is true for marine organisms including sponges, tunicates, asteroids, holothurians, and opisthobranch mollusks. In particular, the so-called “sea slugs” (Mollusca: Gastropoda: Opisthobranchia) provide some of the most remarkable, and best documented, examples of the evolution of chemical defense in marine organisms [3]–[5]. With the regression of the external shell and less reliance upon mechanical defense, chemical defense has played a primary role in the evolution of these animals, which includes also the evolution of systems that facilitate the effective deployment of the defensive metabolites against their predators. Sea hares protect themselves using both passive mechanisms, in which the defensive chemicals are present in the distasteful surface of the skin, and active mechanisms, in which the chemicals are released from specialized glands in response to predatory attacks [6]. Aeolid nudibranchs are remarkable for their “cleptocnidae” – stinging capsules that are expropriated from the cnidarians upon which these nudibranchs feed, and are turned against the slugs’ predators [7]. In chromodorid nudibranchs a general trend is apparent towards the concentration of dietary metabolites in exposed parts of the body (Figure 1) in structures called mantle dermal formations (MDFs), a term that is noncommittal about their function. These structures may have plausibly evolved as mechanisms for concentrating and storing metabolites until releasing them at the appropriate time. However, alternative reasons for the evolution of the MDFs in dorid nudibranchs have been proposed. In particular, it has been suggested that the defensive function of these structures is only secondary and that they evolved primarily for another function such as avoiding autotoxicity [8]. This evolutionary scenario is based on the assumption that the localization of defensive compounds is not essential for defense. There has even been proposed a scenario in which the MDFs in effect originated as a sort of kidney and only later evolved into a defensive mechanism [9]. Herein we present some empirical evidence, produced by recently proposed methods [10], [11], that is germane to such issues. In particular, we consider data on the family Chromodorididae (order Nudibranchia). These often very colorful animals are noteworthy for their ability to sequester defensive metabolites from sponges [3], [4], [12], [13]. The basic interpretation of evolution in this family has been that metabolite-containing storage reservoirs, which are located in restricted and easily accessible areas of the mantle, have a defensive role [14]–[23], while the transfer of defensive diet-derived metabolites to the MDFs of a chromodorid nudibranch has been proved experimentally [19]. However, although feeding specificity within the Chromodorididae has been extensively studied with special attention to the metabolites isolated from both the nudibranchs and their sponge prey [24], and although many relevant data are now available on the histology and putative ecological significance of the MDFs [9], debate continues about how the metabolites of the MDFs are released or come into contact with potential predators. These structures normally lack a duct or duct system [15]. Even though a duct leading to the exterior has been shown to occur in a few cases within the Chromodorididae, in most cases the mechanism for exudation of the contents of the glands remains unclear. The situation is thus quite different from that in other opisthobranchs. In sea hares, for example, the defensive chemicals are present on the body surface or are secreted upon predatory attack [6]. In each chromodorid genus the MDFs are arranged in a characteristic way [25], and these arrangements, along with the characteristic color patterns and mantle shape, all play important roles in the defense of these animals [26]. As is often the case with organisms having chemical defense, examples of aposematism and Müllerian mimicry have been reported [11], [21], [27]. However, it has recently been found that, even in the absence of typical MDFs (as occurs in a few chromodorid species), distasteful metabolites are accumulated at hitherto unexpectedly high concentrations in the exposed mantle rim of these brightly colored nudibranchs [11]. In some lineages a tendency towards a reduction in parts of the mantle has been noted. This reduction is particularly evident in the genus *Ceratosoma*, where the mantle skirt is restricted to a few lobes including a large dorsal horn containing MDFs (Figure 1F). The observation that the dorsal horn is frequently damaged suggests that the attention of predators has been directed to a distasteful part of the body, thereby reducing the amount of damage to the slug [26], [28]. This hypothesis was further supported in *C. gracillimum* and *C. trilobatum* by the presence of a feeding deterrent sesquiterpenoid, mainly concentrated in the MDFs of the dorsal horn [29]. However, this suggestion remained controversial given the small sample size and because neither quantitative nor statistical treatment of data was provided.

The existing chemo-ecological literature routinely presents efforts to assess the palatability of nudibranchs and their metabolites to predators, but such efforts rarely provided realistic data on the natural concentration of the metabolites in the various parts of the body, and give uncertain results because degradation can occur during chromatography. Moreover, they have not adequately evaluated the activity of the different compounds as they affect the predators. New techniques for measuring the levels of metabolites and new ways of evaluating their dose-dependent impact on model predators now make it possible to provide meaningful quantitative data that are relevant to such issues.

We studied nudibranchs of six species belonging to five genera of the family Chromodorididae (Figure 1), collected in the South China Sea. The distribution of metabolites isolated from these species and their feeding deterrent activity have been evaluated to provide, in a synthesis with field observations, a better understanding of the role of confined high concentrations of dietary compounds in the most exposed parts of the mantle of chromodorid nudibranchs.

## Materials and Methods

### Animals

Specimens of the nudibranchs *Glossodoris atromarginata* (3 individuals, average size 30 mm), *Chromodoris sinensis* (3 individuals, average size 20 mm), *Hypselodoris* sp. (3 individuals, average size 15 mm), and *Risbecia tryoni* (3 individuals, average size 40 mm), were collected along the coast of Whei Zhou Island (South China Sea, China), while *Hypselodoris infucata* (3 individuals, average size 20 mm), and *Ceratosoma gracillimum* (3 individuals, average size 90 mm), were collected at the South coast of Hainan Island (South China Sea, China). The sampling activities were carried out by SCUBA diving in dive sites for which no specific permits were required, and they did not involve endangered or protected species. All samples were frozen immediately after collection and stored at −20°C until their chemical analysis.

### Dissection and Extraction

Each nudibranch specimen was dissected into four parts: 1) inner organs (viscera), 2) mantle tissue devoid of MDFs, 3) MDFs, and 4) dissection residuals. The volume of the first three dissected parts was measured for each individual in acetone by displacement of the solvent in graduated glass tubes or micro syringes depending on their size. The obtained volumes of a given number of spheroidal MDFs from *R. tryoni* and *C. gracillimum* were also compared as a validation with the calculated volumes of spheroids with the same axes, just observing minute variations near to our limit of detection in the glass microsyringes (data not shown). Afterwards all dissected parts were extracted separately with acetone by crumbling the tissue with a small glass mortar and pestle and then treating it with ultrasound vibration for 1 minute. The extraction was repeated four times for each sample. After concentration in a rotary evaporator, residual water was removed by extraction with diethyl ether.

During dissection of the nudibranchs, the relative hardness of MDFs was also evaluated by applying various degrees of pressure with a blunt dissection probe. The test was carried out on very small tissue samples and photographs were taken under light microscope on unmounted slides, by placing a drop of water over each sample. The samples were not re-used for the evaluation of the natural concentration of the metabolites, but combined with the dissection residuals.

### Anatomical Distribution of the Compounds

The chloroform-soluble part of each extract was separately subject to ^1^H NMR quantification by adding a known amount of dimethylfumarate (Sigma Aldrich, Germany) as an internal standard. ^1^H NMR spectra were recorded on a Bruker Avance 400 MHz. The dimethylfumarate signals at *δ* 6.86 in the ^1^H NMR spectra of the crude extracts, resulting from two protons, were used for integration to quantify the different metabolites in comparison with their diagnostic signals, by using the Bruker software package (Bruker, BioSpin GmbH, Germany), as follows:
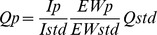
where *Qp* is the amount of the compound in mg, *Ip* is the integral value for a single proton of the compound, *Istd* is the integral value for a single proton of the internal standard, *EWp* is the equivalent weight of the compound (molecular weight), *EWstd* is the equivalent weight of the internal standard, and *Qstd* is the amount in mg of the internal standard.

Natural volumetric concentrations were determined by dividing the calculated amount of each compound by the respective tissue volume (mg/ml). Mean and standard errors were calculated for each tissue part.

### Purification and Identification of Metabolites

**Figure 2:**
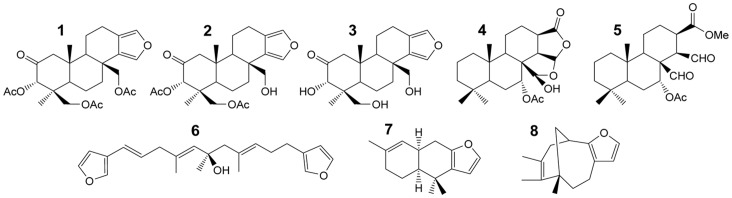
Structures of compounds 1–8.

After registration of the crude mixture NMR spectra, the extracts from the different parts of each species were combined. Each of the combined extracts was chromatographed on a silica gel column (Merck Kieselgel 60 powder) packed with *n-*hexane and eluted with a gradient of *n*-hexane/diethyl ether to give pure compounds and semi-purified mixtures. The mixtures were further separated by HPLC equipped with a Kromasil C18 column (Phenomenex, 5 µm, 250×10.00 mm) using a gradient from 70∶30 methanol/water to 100% methanol over 30 min, monitored by measuring absorbance at 210 nm. Purified compounds **1**–**4** and **6**–**8** (Figure 2) were identified by ^1^H and ^13^C NMR by comparison to published data [30]–[35]. The dialdehyde **5** was not purified, because of its easy conversion into the corresponding cyclic hemiacetal **4** during the purification procedures. Therefore, its identification was performed by comparing the NMR data recorded on the crude extract from the *C. sinensis* MDFs with those reported in the literature [35], [36]. NMR spectra were acquired in CDCl_3_ on a Bruker DPX-300 MHz, a Bruker Avance 400 MHz and a Bruker DRX-600 MHz.

### Feeding Deterrence Assay

The compounds were tested for their feeding deterrence activity against the common trophic generalist shrimp *Palaemon elegans* (Rathke, 1837), which is not an endangered species. This model was used to assay and compare responses to different metabolites at various dosages. Assays were performed as described in Mollo et al. [10], by using food pellets treated with the different compounds at concentrations ranging from 1 to 6 mg/ml. Each pure compound dissolved in 0.5 ml of acetone was added to a mixture of alginic acid (30 mg), ground freeze-dried squid mantle (50 mg), and purified sea sand (30 mg; granular size 0.1–0.3 mm). After evaporation of the solvent, one drop of food coloring (E124 and E110) and distilled water was added to give a final volume of 1 ml. Food coloring was added for easy detection of the ingested food in the digestive tract of the shrimps. The mixture was stirred, loaded into a 5 ml syringe, and extruded into a 0.25 M calcium chloride solution for 2 min to harden. The resulting spaghetti-like red strand was cut into 10 mm long pellets. Control foods were made in the same manner, with the addition of 0.5 ml of acetone but without the purified metabolites. The shrimps were collected in sampling locations not privately owned or protected in any way along the coast of Pozzuoli, Italy, and habituated to the control food in captivity for a week before experiments. After three days of fasting, ten randomly picked shrimps were assayed as a series of individual replicates for each concentration and the control (n = 10 for each series). Shrimps were placed individually into 500 ml plastic beakers filled with 300 ml of seawater. A colored food strip was given to each shrimp, and shrimps were not reused. For each tested compound, control and treatments were carried out in parallel (8 control series in total). The presence of a red spot visible by transparency in the gastric mill and the stomach of the shrimps after 30 min was considered as acceptance of food, while the absence of the spot gave a rejection response (Figure S1). Statistical analysis between treatments and controls was performed using the two-tailed Fisher-Exact test, with α = 0.05 as significant level.

After the experiments, the shrimps were returned to the field at the same locations where they were collected.

### Evaluation of the Shrimps’ Ability to Produce Damage to Mollusks

Models of unprotected nudibranchs were sculptured in squid muscle to reproduce the body shape of both a *Hypselodoris*- or *Risbecia*-like nudibranch with a small mantle skirt, and a *Ceratosoma* nudibranch with its dorsal horn. The two models were placed on a terracotta tile, together with another one devoid of any mantle skirt, and waiting for 5 minutes allowed their adhesion. The tile with the models was then placed in a seawater aquarium in the presence of twelve *P. elegans* shrimps (after two days fasting). The whole experiment was repeated three times, and the damage produced on the models by the shrimps was recorded after 60 minutes.

## Results

### Isolated Compounds and their Anatomical Distribution

In the chemical part of this study, we identify the known sponge-derived metabolites **1**–**8** (Figure 2), and assessed their natural volumetric concentration in three different anatomical parts of the nudibranchs obtained by dissection: inner organs (viscera), mantle tissue fragments devoid of MDFs (mantle), and MDFs (Table 1). Only in the case of *Chromodoris sinensis* the ramifying structure of the MDFs, which are widespread along the entire mantle rim (Figure 1B), prevented a precise dissection; they were dissected along with a small part of the mantle edge. Compared to the levels in other body parts, all metabolites, except compound **3**, reached by far the highest concentrations in the mantle glands.

**Table 1:** Anatomical distribution of the metabolites.

Species	Compound	Viscera (mg/ml)	Mantle (mg/ml)	MDFs (mg/ml)
***Glossodoris atromarginata***	**1**	1.6±0.5	1.3±0.2	(0.26±0.07)×10^2^
	**2**	1.1±0.1	0.8±0.1	(1.04±0.05)×10^2^
	**3**	7.5±1	7.2±0.7	below 0.5
***Chromodoris sinensis***	**4**	14±8	9±3	(1.5±0.3)×10^2^
	**5**	n.d.	below 0.5	(4.1±0.3)×10^2^
***Hypselodoris sp.***	**6**	8±2	4.2±0.6	(7.7±0.7)×10^2^
***Hypselodoris infucata***	**7**	1.2±0.1	1.2±0.1	(1.9±0.2)×10^2^
***Risbecia tryoni***	**7**	1.2±0.5	1.1±0.2	(3.5±0.1)×10^2^
***Ceratosoma gracillimum***	**7**	1.1±0.1	below 0.5	(3.7±0.2)×10^2^
	**8**	below 0.5	below 0.5	(4.6±0.3)×10^2^

Natural volumetric concentrations (mg/ml anatomical section) of compounds **1**–**8** were quantified by NMR, in viscera, mantle, and MDFs of the studied nudibranch species. Mean concentrations from three individuals ± SEM are presented. Trace concentrations below 0.5 mg/ml are not reported. n.d., not detected.

### Feeding Deterrence Activity

**Figure 3:**
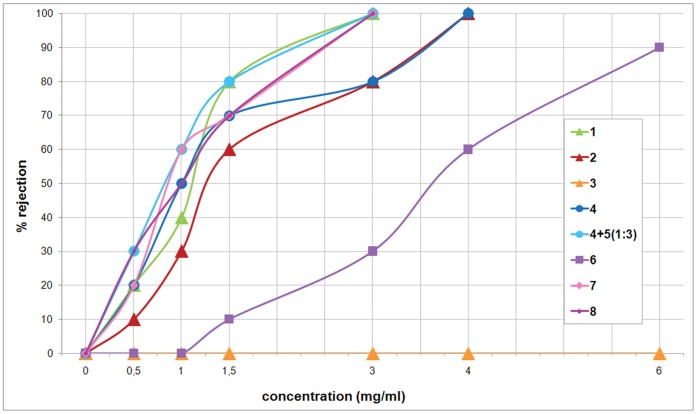
*Palaemon elegans* alimentary response. Dose-response curves obtained by offering food pellets treated with purified compounds **1**–**4** and **6**–**8** to shrimps, at volumetric concentration ranging from 0.5 to 6.0 mg/ml. Instead of compound **5**, which we have been unable to isolate, we assayed the crude extract containing a 1∶3 mixture of **4** and **5**. The zero concentration was defined as control (8 control series in total) and significant differences in the consumption of treated vs. control pellets have been evaluated by two-tailed Fisher’s exact test (α = 0.05, n = 10 for each compound concentration tested). *P* values lower than 0.05 are as follows: *P* = 0.03251, *P* = 0.01084, *P* = 0.00310, *P* = 0.00071, *P* = 0.00012, *P* = 0.00001, respectively for 50%, 60%, 70%, 80%, 90%, and 100% rejection.

The purified compounds were tested for their activity as a feeding deterrent against the trophic generalist shrimp *Palaemon elegans.* As summarized in Figure 3, all purified compounds, except compound **3**, were significantly active at concentrations ranging from 1 to 4 mg/ml (*P* values are listed in the figure legend).

Combining these results with the data on the anatomical distribution of the metabolites (Table 1), we obtained the following information, listed by species.

*Glossodoris atromarginata* (Cuvier, 1804). The quantification of the metabolites revealed that the feeding deterrent furanospongianes spongiatrioltriacetate (**1**) and spongiatriol-diacetate (**2**) [30], [31] are accumulated in the MDFs. Both compounds resulted in significant activity as feeding deterrents at concentrations much lower than those detected in the mantle reservoirs. As a comparison between closely related metabolites, we also isolated compound **3**, spongiatriol [30], especially distributed in the mantle and viscera of *G. atromarginata*. This compound, however, did not show significant activity in feeding deterrence at the assayed concentrations.

*Chromodoris sinensis* Rudman, 1985. This species has never been chemically investigated previously. The crude extract from the border of the mantle, which includes the MDFs, contained a 1∶3 mixture of compounds **4**, aplyroseol-2 [35], and its corresponding dialdehyde **5** [36]. Given that we did not isolate the pure compound **5**, because of its easy transformation into the hemiacetal **4** during chromatography, the activity of purified **4** was compared to the crude extract, where **5** represented the main metabolite. The feeding deterrent activity of the mixture was more deterrent relative to the pure compound **4** at all assayed concentrations, reasonably attributable to the higher activity of the dialdehyde **5**. Compound **5** was absent in the viscera of *C. sinensis*, thus suggesting its possible origin by enzymatic transformation and activation of metabolite **4** during its transfer to the MDFs.

*Hypselodoris* sp. A highly significant level of feeding deterrent activity was recorded at relatively high concentrations (6.0 mg/ml) for compound **6**, (+)-tetradehydrofurospongin-1 [32], but the metabolite concentration exceeds the extremely high value of 700 mg/ml in the MDFs of this unidentified chromodorid species of the genus *Hypselodoris* Stimpson, 1855.

*Hypselodoris infucata* (Ruppell & Leuckart, 1830 or 1831), and *Risbecia tryoni* (Garrett, 1873). We only found compound **7**, (−)-furodysinin [33], in the MDFs, showing highly significant feeding deterrent activity at concentrations much lower than those detected in the MDFs.

*Ceratosoma gracillimum* Semper in Bergh, 1876. We found both compound **7**, (−)-furodysinin [33], and compound **8**, nakafuran-9 [34], in the MDFs. Also compound **8** showed highly significant feeding deterrent activity at relatively low concentrations.

### Relative Hardness of MDFs

When we evaluated the relative hardness of the MDFs, by applying various degrees of pressure during dissection of the nudibranchs, we observed that the low level of rupture energy that we applied with a dissection probe (visible in Figure S2b) to the MDFs was enough to force the release of their content. MDFs of *G. atromarginata* (Figure S2a,b,c) were easily broken, resulting in the release of what seem to correspond to the “vacuoles” described by Wägele et al. 2006 [6]. An easy release of similar structures was also observed in *C. sinensis*. Slightly higher pressure was required to break the MDFs of *H. infucata* (Figure S2d), *Hypselodoris sp.,* and *C. gracillimum* (Figure S2e), allowing the release of "lipophilic drops". The highest rupture energy was required for *R. tryoni*, due to the greater thickness of the external membrane of its MDFs (Figure S2f). However, it was obviously lower than the forces that could be generated by the predators’ claws, teeth or radulae. This shows that the damage of the mantle edge produced by a predator attack can evoke the release of an enormous amount of repellent lipophilic metabolites.

### Shrimps’ Preference for Shape

When we offered mock unprotected nudibranchs made of squid resembling the condition of an uncolored mantle edge and of a dorsal finger-shaped appendage to shrimps (Figure S3), damage to the models was produced selectively in the position where the MDFs in the corresponding nudibranchs are located. This straight-forward experiment was repeated three times with similar results.

## Discussion

The study of the anatomical distribution of defensive metabolites in all chromodorid species here analyzed has made it clear that the unpalatable compounds reach very high concentrations in the MDFs, which are located in the more exposed parts of the body. Our findings also confirm that chromodorids are trophic specialists that derive terpenoids from the sponges they eat. However, two of the species studied seem to diverge from the generalization that each chromodorid genus utilizes a characteristic class of dietary terpenoids [12]. Despite the frequent specialization of *Glossodoris* species on sesterterpenoids (25 carbon atoms) and that of *Hypselodoris* species on sesquiterpenoids (15 carbons), we found diterpenoids (20 carbons) in *G. atromarginata*, and a degraded furano-sesterterpenoid (21 carbons) in *Hypselodoris* sp. In addition, comparing our previous data to present data, intra-specific variations appear in the chemical composition of *G. atromarginata* and *C. gracillimum* [29], [37], [38]. These observations suggest that there is still much to learn about the evolution of food specialization in this family, especially because a recent review suggests that many apparent anomalies could be due to the misunderstanding of either sponge or chromodorid phylogeny [24]. Be this as it may, our results indicate that only predator-deterring compounds are accumulated and stored in the MDFs. This, together with the reduction of the mantle skirt and changes of body shape, supports the hypothesis that these animals direct the attention of predators to distasteful and sacrificial regions of their body that are distant from the vital organs thereby avoiding serious damage [28]. According to this view, when disturbed, the chromodorid nudibranchs remain in place, retracting rhinophores and gills (Figure S4), which are respectively chemosensory and respiratory organs that cannot be sacrificed because of their crucial importance for the survival of the animal, and deploy the sacrificial parts of the mantle against the predators.

Fish, crustaceans and other animals are known to attack opisthobranchs. We used the marine decapod *P. elegans* as model predator because of both its easy availability near to the chemical laboratory where the bioassays were carried out, and its broad adaptability, allowing its survival for long time in a small volume of seawater. Although this species does not co-occur in the same area where the studied nudibranchs were collected, generalist palaemonids are widespread and very common, including in the South China Sea. We did not attempt field experiments owing to such practical difficulties as assessing the state of conservation and the purity of the compounds immediately prior to the assays in remote locations. There were, however, additional reasons why we focused on one predator instead of a range of sympatric fishes and invertebrates. The use of a non-local species for the feeding experiments allowed us to exclude confounding effects due to avoidance-learning. Furthermore, we were limited in the amount of available compounds and given our interest chose to test different concentrations against a single predator rather than testing low concentrations against a number of predators. Finally, we chose a model predator that can be monitored when attacking model nudibranchs (i.e. in fish the prey would be taken up as a whole and processes in the mouth cavity could not be observed). As expected, when we offered models of unprotected nudibranchs made of squid to these shrimps (Figure S3), damage was produced on the more accessible parts of the mantle where the MDFs in the corresponding true nudibranchs are located. This can be explained from a mechanical point of view: shrimps more easily break thin and exposed parts of the nudibranch bodies, while a flat surface without protrusions is evidently difficult to attack, at least by small predators. This simple experiment confirms the validity of the overall experimental design, showing that the chosen model predator can eat unprotected mollusks, and that anatomical parts containing the highest level of defensive metabolites in the actual animals are the first to be attacked. Given that so little rupture energy is required to break the MDFs (Figure S2), a similar attack, on real nudibranchs in field, would have caused the release of an enormous amount of repellent lipophilic metabolites to interact locally with chemosensory systems.

**Figure 4:**
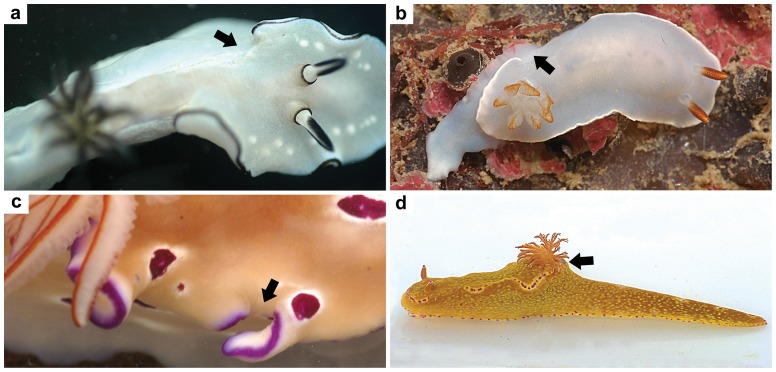
Damage to the mantle. Photos of living individuals of (**a**) *G. atromarginata*, (**b**) *Hypselodoris* sp., and (**c**) *R. tryoni* showing various degree of amputations and healing at the border of the mantle (indicated by arrows). Photo **d** shows an individual of *Ceratosoma tenue* in which the whole dorsal horn has been cut off, probably bitten off.

Adaptation is an historical concept, and is best studied in the context of a phylogenetic analysis that is combined with other evidence. Physiology and functional anatomy are often invoked as evidence that the metabolites are presently deployed as adaptive scenarios suggest. Chemical data play an important role in such research, and the present study invokes such reasoning. Subsequent to their common ancestry the chromodorid nudibranchs have become adapted so as to defend themselves in a wide range of habitats and against a variety of predators. However, there is reasonably good evidence of how the still extant predators and prey interact in the field. In the field one often encounters chromodorid nudibranchs with the mantle border damaged but nonetheless having survived the attacks that inflicted these wounds. The images in Figure 4 are examples. This confirms that the defensive mechanism described does operate in nature. Even though this is a small sample, our observations of analogous phenomena occurring in many other chromodorid species in their own habitats worldwide strongly support the general rule that the chromodorids' MDFs are positioned so as to be preferentially attacked by predators, in a way that maximizes their impact on chemosensory organs. The metabolites are lipophilic and not released into the surrounding seawater, but rather in the mouth of the potential predators, increasing the local concentration and enhancing the defensive effect as well as the likelihood of the predators learning to avoid these prey items. In addition, the localization and high level of feeding deterrent compounds may have such economic advantages as optimizing resource allocation, given that a considerably larger amount of the compounds would need to be accumulated throughout the body to reach the same concentration that occurs in the MDFs. Our results indicate that only feeding deterrent compounds are accumulated in the MDFs, and other data reported in the literature support this as a general rule [3], [12], [13]. Interestingly, the case of *G. atromarginata* suggests that the defensive metabolites stored in the MDFs can be selectively accumulated even discriminating between closely related compounds. This is supported by the comparison of ^1^H NMR spectra from different body parts of each studied species (Figure S5, S6, S7, S8, S9, S10) showing that other lipid metabolites and steroids are present in both viscera and mantle crude extracts, which almost completely disappear in the crude extract from the MDFs. Apparently, the extreme concentrations observed in the case of *Hypselodoris* sp. are due to the MDFs being full of the dietary compound **6**, whereas the layer surrounding such reservoirs contributes very little to their volume.

Avila and Paul found that predators were less likely to eat nudibranchs (*Glossodoris pallida*) in which the mantle borders had been left intact, than they were to eat conspecifics in which the mantle borders had been trimmed away [8]. That is very much what one would expect if the defensive metabolites are in fact concentrated in a place that maximizes their efficacy. However, their experiments with artificial diets showed that it did not matter whether the metabolites were distributed uniformly throughout the food cubes, or concentrated along the border. On this basis they concluded that “localization is not essential for defense but instead perhaps for other physiological purposes such as secreting chemically rich mucus or avoiding autotoxicity”. Leaving aside the problems of interpreting negative results, their suggestion doesn’t agree with the fact that the MDFs of almost all chromodorids lack the sort of ducts that would allow them to function in that fashion. More recently Wägele, Ballesteros & Avila take up the same theme, supporting the idea that the initial role of accumulation structures was that of excretion or autoprotection from the dietary chemicals, and that their defensive function evolved later [9]. This makes the puzzling assumption that for some reason the chromodorids are physiologically able to separate out the metabolites during digestion but are then unable to remove them from the body through normal pathways, and yet are able to transfer and compartmentalize them in the mantle tissue.

Here we show that the localization of high levels of defensive chemical weapons in MDFs located in exposed anatomical structures play a decisive role in chemical defense even in absence of an active secretion. The presence of the metabolites at low concentration on the mantle surface could represent a first line of chemical defense, providing a somewhat repulsive taste, with a mode of action similar to that of the compounds that are distributed over the body surface of many other organisms, including toads, frogs and salamanders [39]. However, the breakage of the MDFs, occurring during the more determined and harmful attacks on chromodorid nudibranchs, allows the release of a huge quantity of highly repellent lipophilic metabolites that interact locally with chemosensory systems. Such a massive dose represents the *extrema ratio*, an extreme solution after an animal or part of it is in the predator’s mouth.

By including a historical/evolutionary time dimension in the discussion, we propose a plausible diachronic scenario, capable of explaining whether, over a long series of generations, localization has been favored by natural selection. According to the “pre-adaptive” scenario first suggested by Faulkner and Ghiselin [5], a shelled ancestor took to feeding upon chemically defended prey organisms, incidentally becoming repugnant to predators. This innovation set the stage for the elaboration of chemical defense and the reduction of the shell. It was the basis for an adaptive radiation, in which the various lineages have diversified both their diets and their utilization of defensive metabolites. On the other side, with the regression of the external shell and its role in mechanical defense, the primitive function of the mantle of producing the shell has been lost. This has led to a reduction in the size of the mantle within the Chromodorididae, with the conservation of easily accessible mantle edges and projections, where extremely high doses of distasteful compounds are actually localized. We propose that this localization is the result of the evolutionary optimization of resource allocation, with the accumulation of progressively increasing concentrations of distasteful metabolites in exposed sacrificial body parts.

The legend of the Trojan horse that allowed the Greeks to get inside the walls of their enemies’ city represents a clever way of packaging and delivering offensive weaponry. As a defensive variant of the same strategic theme, chromodorid nudibranchs offer parts of their bodies to the predators, allowing a slightly-delayed surprise counterattack in the predator’s mouth due to the presence of concealed “gifts”. These consist of distasteful compounds delivered at high localized doses that, according to Paracelsus’ dose response paradigm [40], make for a strong chemical defense. To understand such warfare it helps to pay close attention to how the weaponry is deployed against the attackers (Figures 1, 4 and S4).

## Supporting Information

Figure S1 **Food palatability assay.** Food preparation (a), food presentation to the shrimps in series of individual replicates (b, c), food acceptance (d), and food rejection (e).
(TIF)

Figure S2 **Relative hardness of the MDFs.** MDFs of (a,b,c) *G. atromarginata*, (d) *H. infucata*, (e) *C. gracillimum*, and (f) *R. tryoni*, were broken by a dissection probe (indicated by yellow arrow in b) allowing the release of lipophilic material (indicated by black arrows). Photomicrographs were taken on unmounted slides, with a drop of seawater placed over each tissue. Scale bar, 500 µm.
(TIF)

Figure S3 **Shape preference assay.** Models of “unprotected nudibranchs”, sculptured in squid muscle (A) to reproduce the body shape of a *Ceratosoma* nudibranch with its dorsal horn (B), and a *Hypselodoris*- or *Risbecia*-like nudibranch with a little mantle skirt (C), were placed in a seawater aquarium along with a mantle-lacking model (D), in the presence of 12 shrimps (E). The shrimps produced damage on the mantle of models B and C (see figures F and G, respectively), whereas we were not able to detect any damage after 60 minutes on model D (figure H).
(TIF)

Figure S4 ***Ceratosoma trilobatum*** **.** The photograph shows an individual of *C. trilobatum* in a black and white partial color effect to highlight the anatomical parts mentioned in the text.
(TIF)

Figure S5 **^1^H NMR spectra of** ***G. atromarginata.*** ^1^H NMR spectra (400 MHz) of crude extracts from one individual of *G. atromarginata* in CDCl_3_ containing dimethylfumarate (DMF) as internal standard. Colored bars show the natural volumetric concentration (NVC, mg/ml anatomical section) of compounds **1**, **2**, and **3** in the different body parts of the nudibranch.
(TIF)

Figure S6 **^1^H NMR spectra of** ***C. sinensis.*** ^1^H NMR spectra (400 MHz) of crude extracts from one individual of *C. sinensis* in CDCl_3_ containing dimethylfumarate (DMF) as internal standard. Colored bars show the natural volumetric concentration (NVC, mg/ml anatomical section) of compounds **4** and **5** in the different body parts of the nudibranch.
(TIF)

Figure S7 **^1^H NMR spectra of** ***Hypselodoris*** **sp.** ^1^H NMR spectra (400 MHz) of crude extracts from one individual of *Hypselodoris* sp. in CDCl_3_ containing dimethylfumarate (DMF) as internal standard. Colored bars show the natural volumetric concentration (NVC, mg/ml anatomical section) of compound **6** in the different body parts of the nudibranch.
(TIF)

Figure S8 **^1^H NMR spectra of** ***H. infucata.*** ^1^H NMR spectra (400 MHz) of crude extracts from one individual of *H. infucata* in CDCl_3_ containing dimethylfumarate (DMF) as internal standard. Colored bars show the natural volumetric concentration (NVC, mg/ml anatomical section) of compound **7** in the different body parts of the nudibranch.
(TIF)

Figure S9 **^1^H NMR spectra of** ***R. tryoni.*** ^1^H NMR spectra (400 MHz) of crude extracts from one individual of *R. tryoni* in CDCl_3_ containing dimethylfumarate (DMF) as internal standard. Colored bars show the natural volumetric concentration (NVC, mg/ml anatomical section) of compound **7** in the different body parts of the nudibranch.
(TIF)

Figure S10 **^1^H NMR spectra of** ***C. gracillimum.*** ^1^H NMR spectra (400 MHz) of crude extracts from one individual of *C. gracillimum* in CDCl_3_ containing dimethylfumarate (DMF) as internal standard. Colored bars show the natural volumetric concentration (NVC, mg/ml anatomical section) of compounds **7** and **8** in the different body parts of the nudibranch.
(TIF)
