# Supplementary figures and images for: Packaging and Delivery of Chemical Weapons: A Defensive Trojan Horse Stratagem in Chromodorid Nudibranchs
Source: PLoS One. 2013 Apr 19;8(4):e62075. doi: 10.1371/journal.pone.0062075 (PMC3631210; doi:10.1371/journal.pone.0062075)

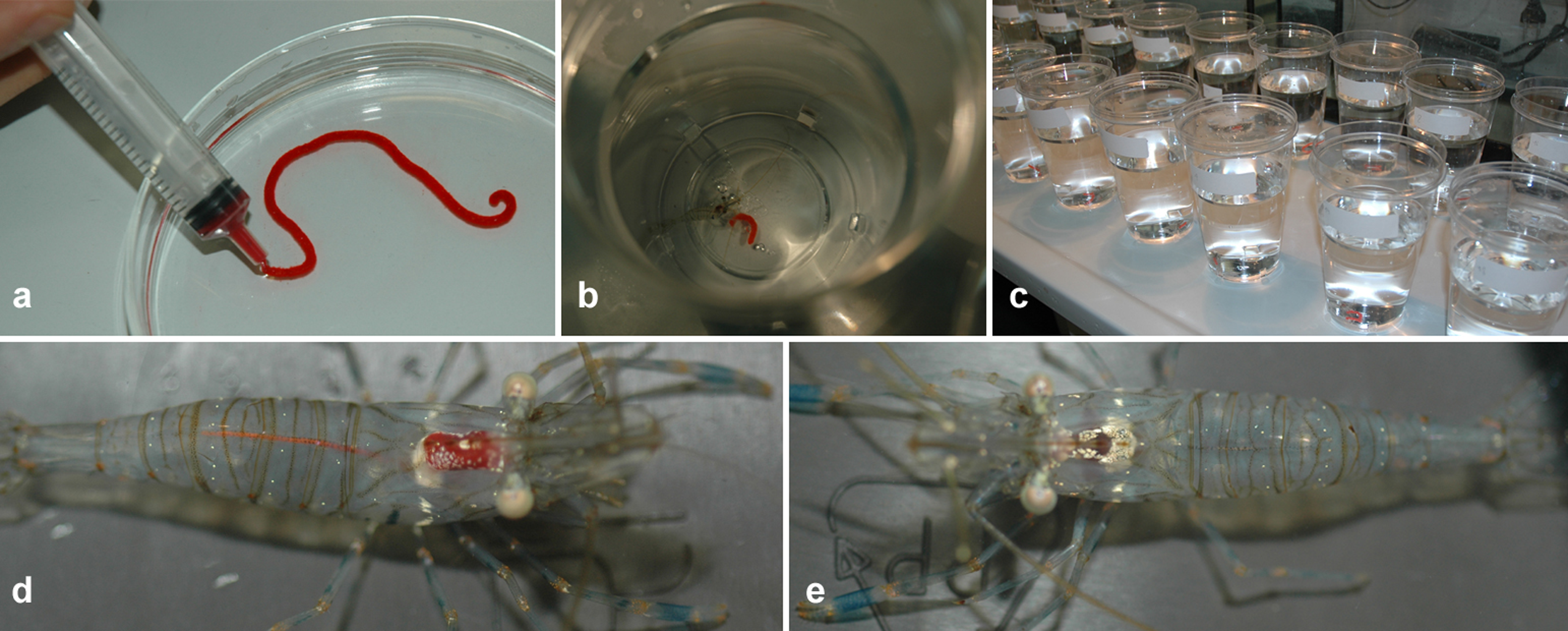

Supplement: Figure S1 — Food palatability assay. Food preparation (a), food presentation to the shrimps in series of individual replicates (b, c), food acceptance (d), and food rejection (e). (TIF) [file pone.0062075.s001.tif]

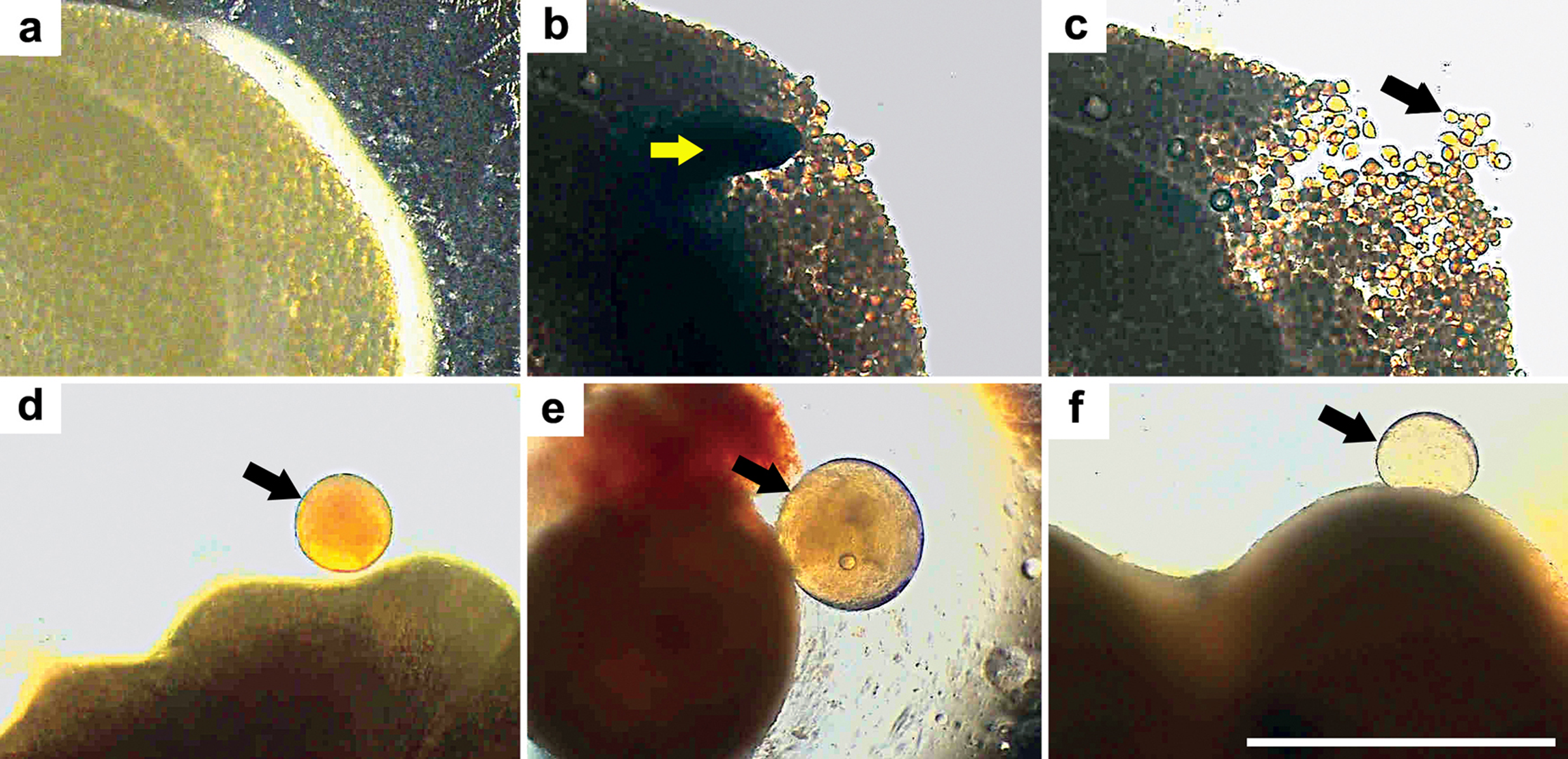

Supplement: Figure S2 — Relative hardness of the MDFs. MDFs of (a,b,c) G. atromarginata, (d) H. infucata, (e) C. gracillimum, and (f) R. tryoni, were broken by a dissection probe (indicated by yellow arrow in b) allowing the release of lipophilic material (indicated by black arrows). Photomicrographs were taken on unmounted slides, with a drop of seawater placed over each tissue. Scale bar, 500 µm. (TIF) [file pone.0062075.s002.tif]

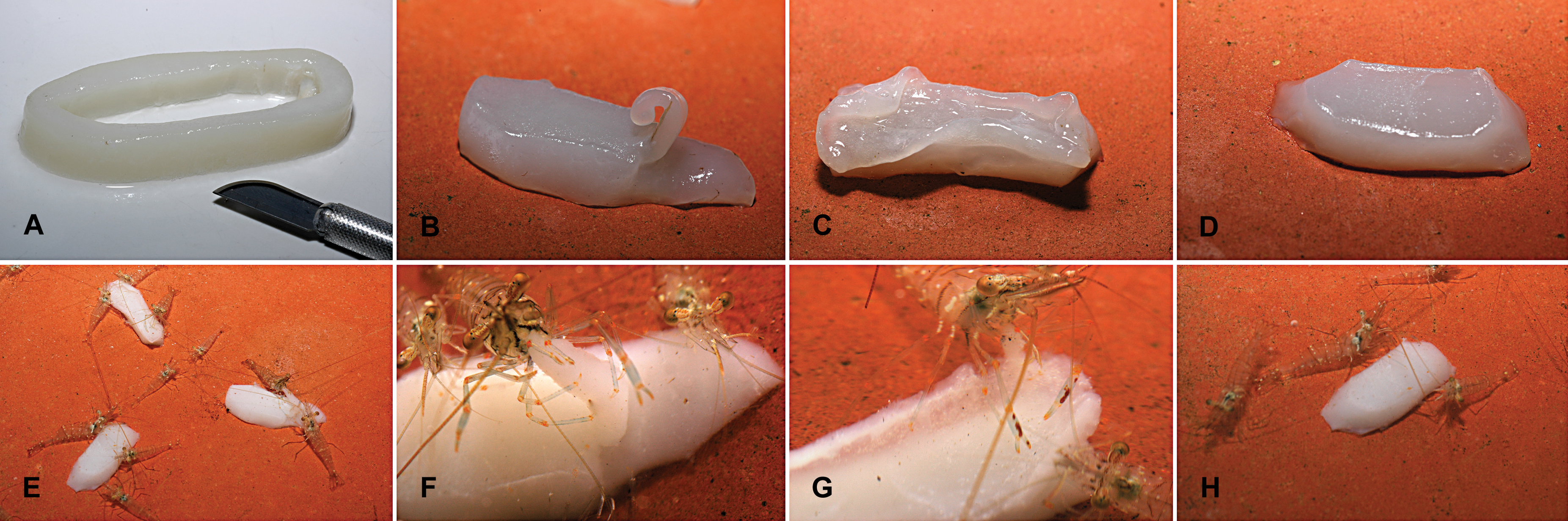

Supplement: Figure S3 — Shape preference assay. Models of “unprotected nudibranchs”, sculptured in squid muscle (A) to reproduce the body shape of a Ceratosoma nudibranch with its dorsal horn (B), and a Hypselodoris- or Risbecia-like nudibranch with a little mantle skirt (C), were placed in a seawater aquarium along with a mantle-lacking model (D), in the presence of 12 shrimps (E). The shrimps produced damage on the mantle of models B and C (see figures F and G, respectively), whereas we were not able to detect any damage after 60 minutes on model D (figure H). (TIF) [file pone.0062075.s003.tif]

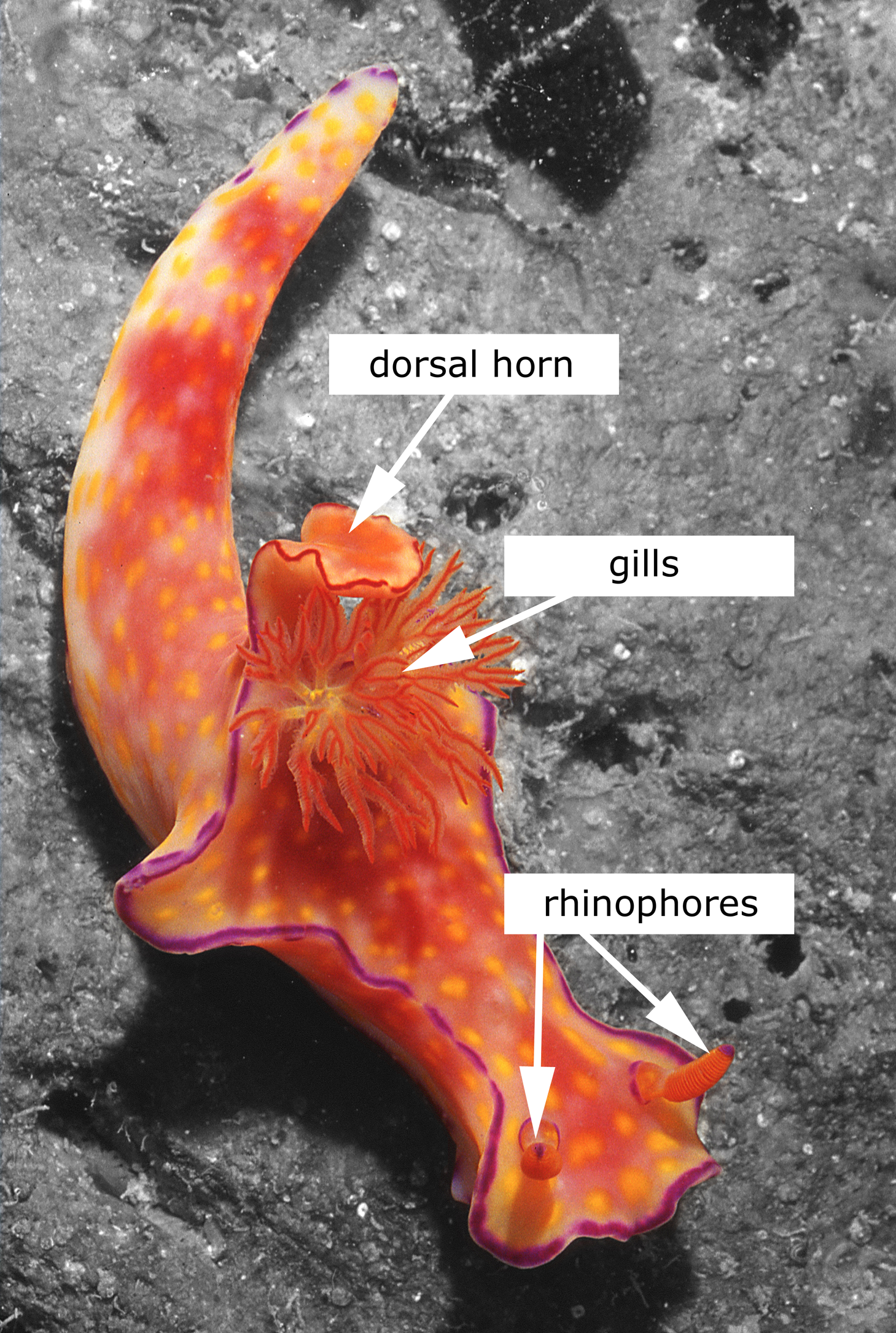

Supplement: Figure S4 — Ceratosoma trilobatum . The photograph shows an individual of C. trilobatum in a black and white partial color effect to highlight the anatomical parts mentioned in the text. (TIF) [file pone.0062075.s004.tif]

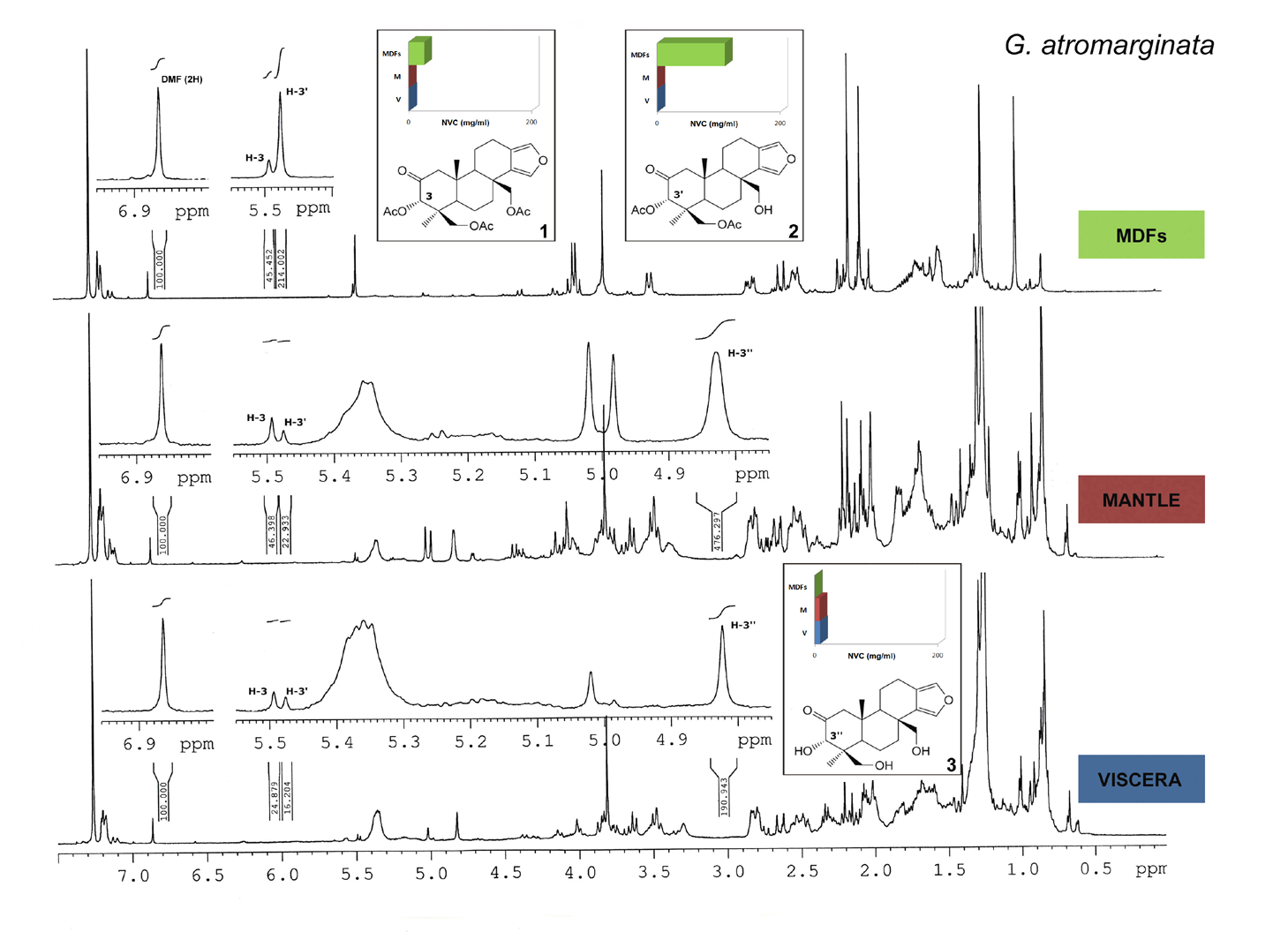

Supplement: Figure S5 — 1H NMR spectra of G. atromarginata. 1H NMR spectra (400 MHz) of crude extracts from one individual of G. atromarginata in CDCl3 containing dimethylfumarate (DMF) as internal standard. Colored bars show the natural volumetric concentration (NVC, mg/ml anatomical section) of compounds 1, 2, and 3 in the different body parts of the nudibranch. (TIF) [file pone.0062075.s005.tif]

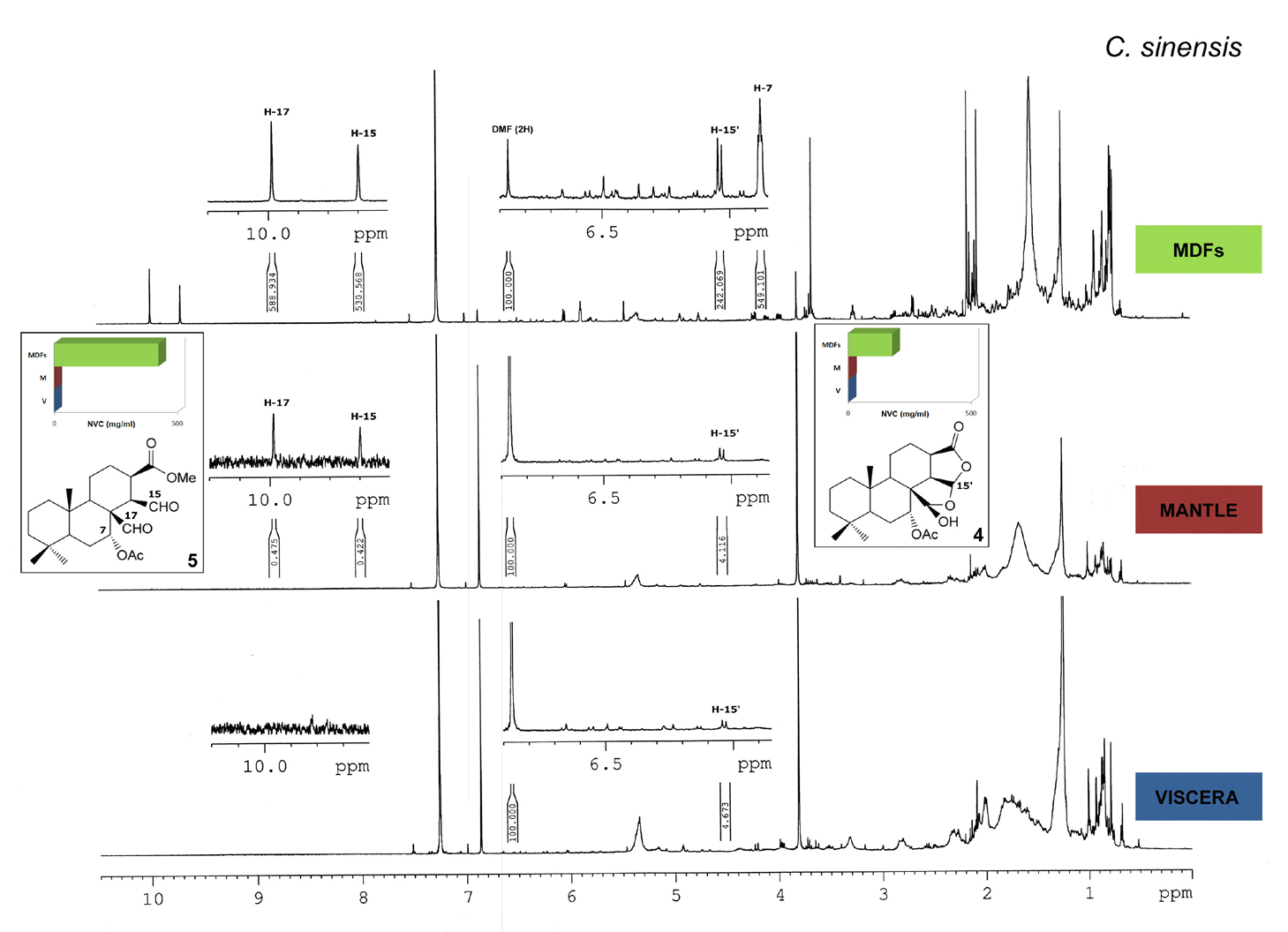

Supplement: Figure S6 — 1H NMR spectra of C. sinensis. 1H NMR spectra (400 MHz) of crude extracts from one individual of C. sinensis in CDCl3 containing dimethylfumarate (DMF) as internal standard. Colored bars show the natural volumetric concentration (NVC, mg/ml anatomical section) of compounds 4 and 5 in the different body parts of the nudibranch. (TIF) [file pone.0062075.s006.tif]

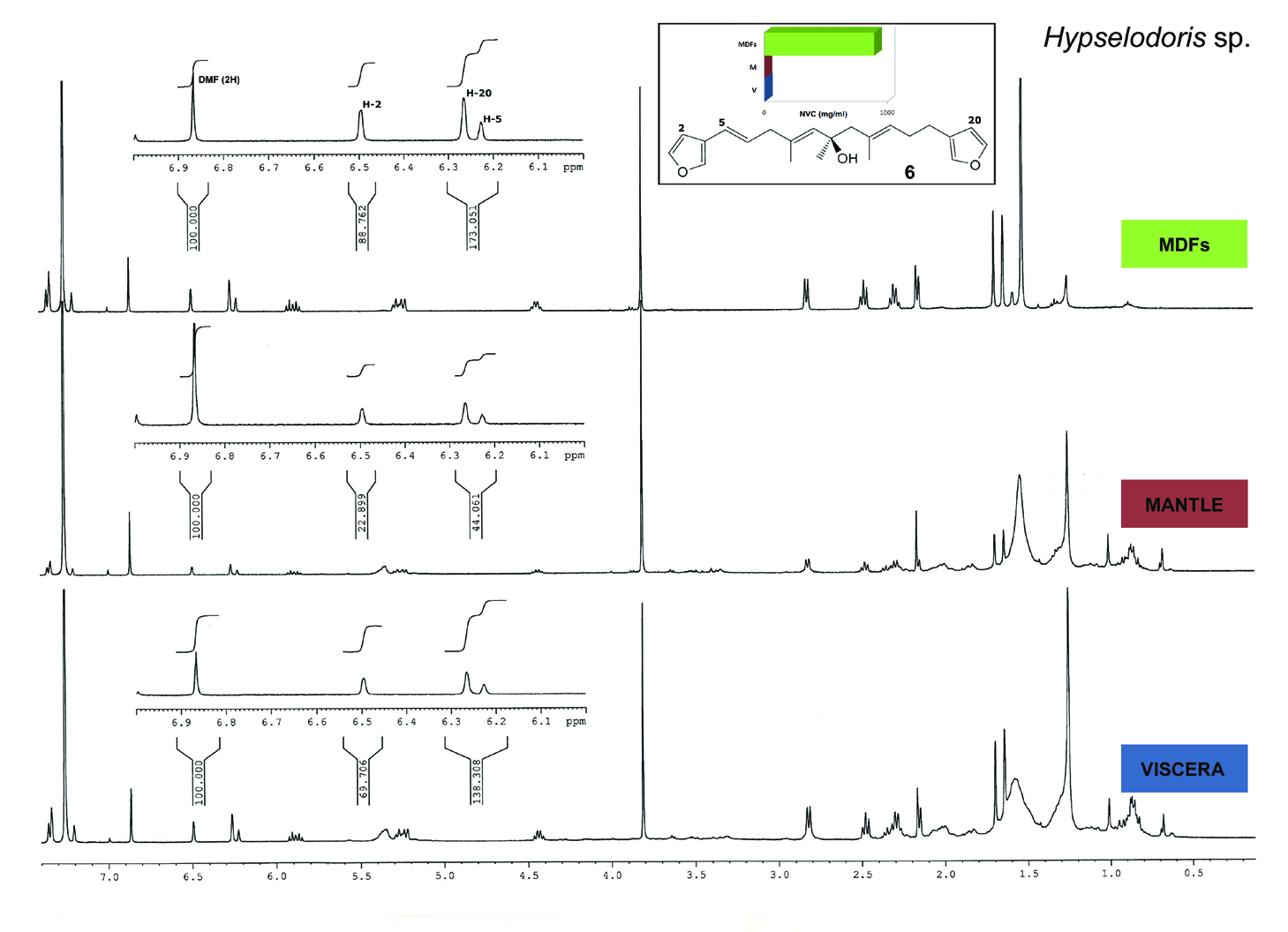

Supplement: Figure S7 — 1H NMR spectra of Hypselodoris sp. 1H NMR spectra (400 MHz) of crude extracts from one individual of Hypselodoris sp. in CDCl3 containing dimethylfumarate (DMF) as internal standard. Colored bars show the natural volumetric concentration (NVC, mg/ml anatomical section) of compound 6 in the different body parts of the nudibranch. (TIF) [file pone.0062075.s007.tif]

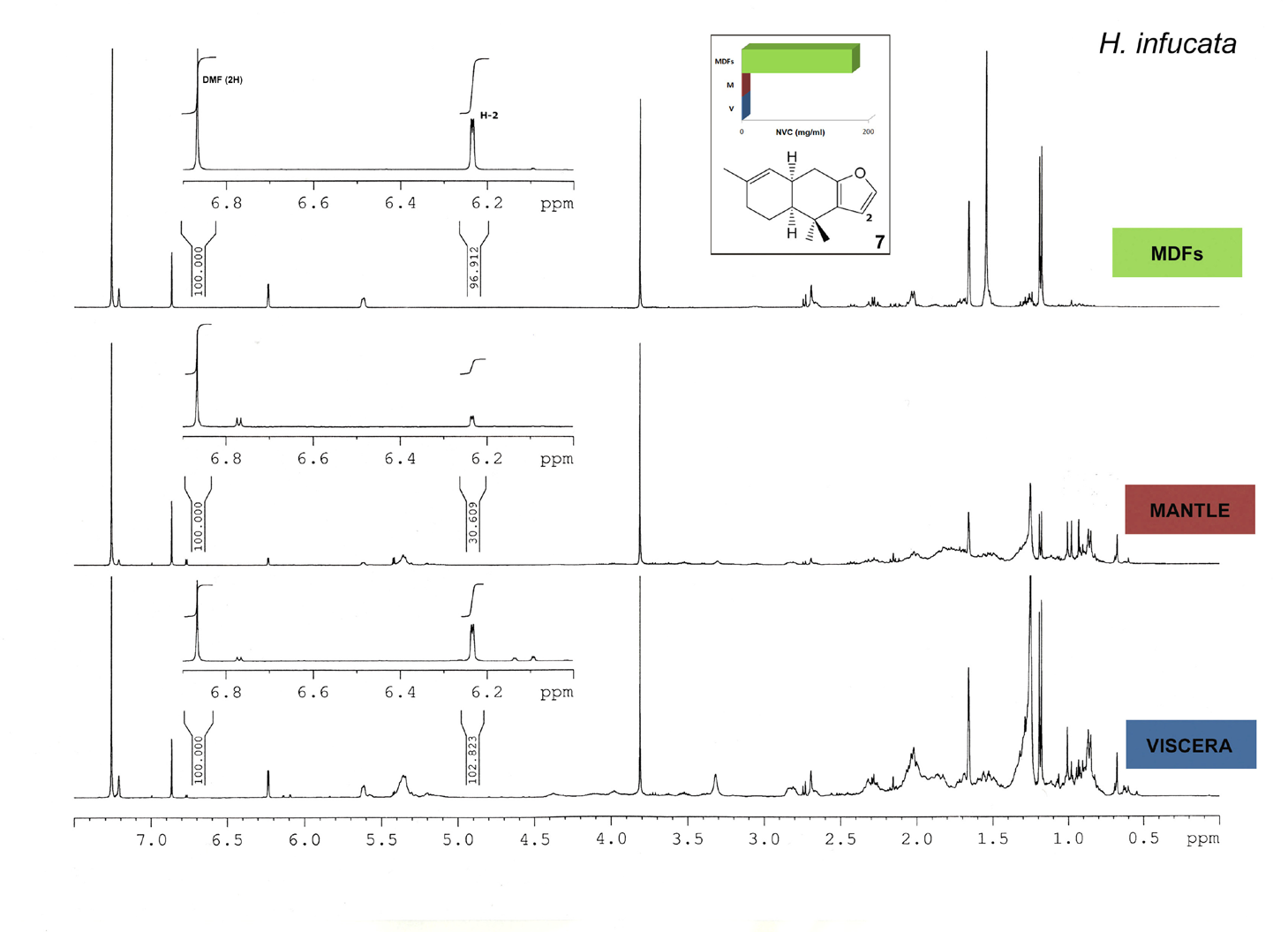

Supplement: Figure S8 — 1H NMR spectra of H. infucata. 1H NMR spectra (400 MHz) of crude extracts from one individual of H. infucata in CDCl3 containing dimethylfumarate (DMF) as internal standard. Colored bars show the natural volumetric concentration (NVC, mg/ml anatomical section) of compound 7 in the different body parts of the nudibranch. (TIF) [file pone.0062075.s008.tif]

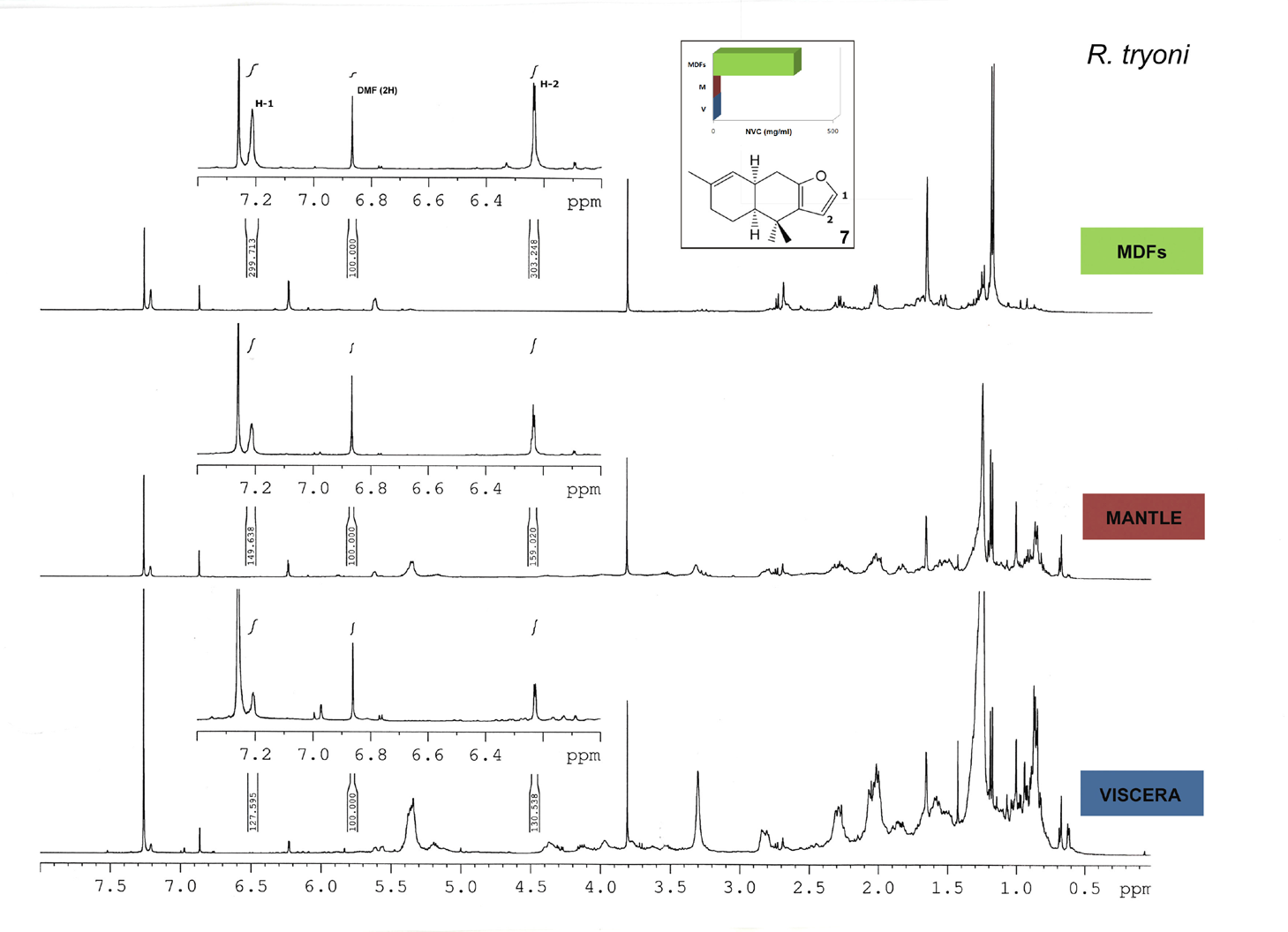

Supplement: Figure S9 — 1H NMR spectra of R. tryoni. 1H NMR spectra (400 MHz) of crude extracts from one individual of R. tryoni in CDCl3 containing dimethylfumarate (DMF) as internal standard. Colored bars show the natural volumetric concentration (NVC, mg/ml anatomical section) of compound 7 in the different body parts of the nudibranch. (TIF) [file pone.0062075.s009.tif]

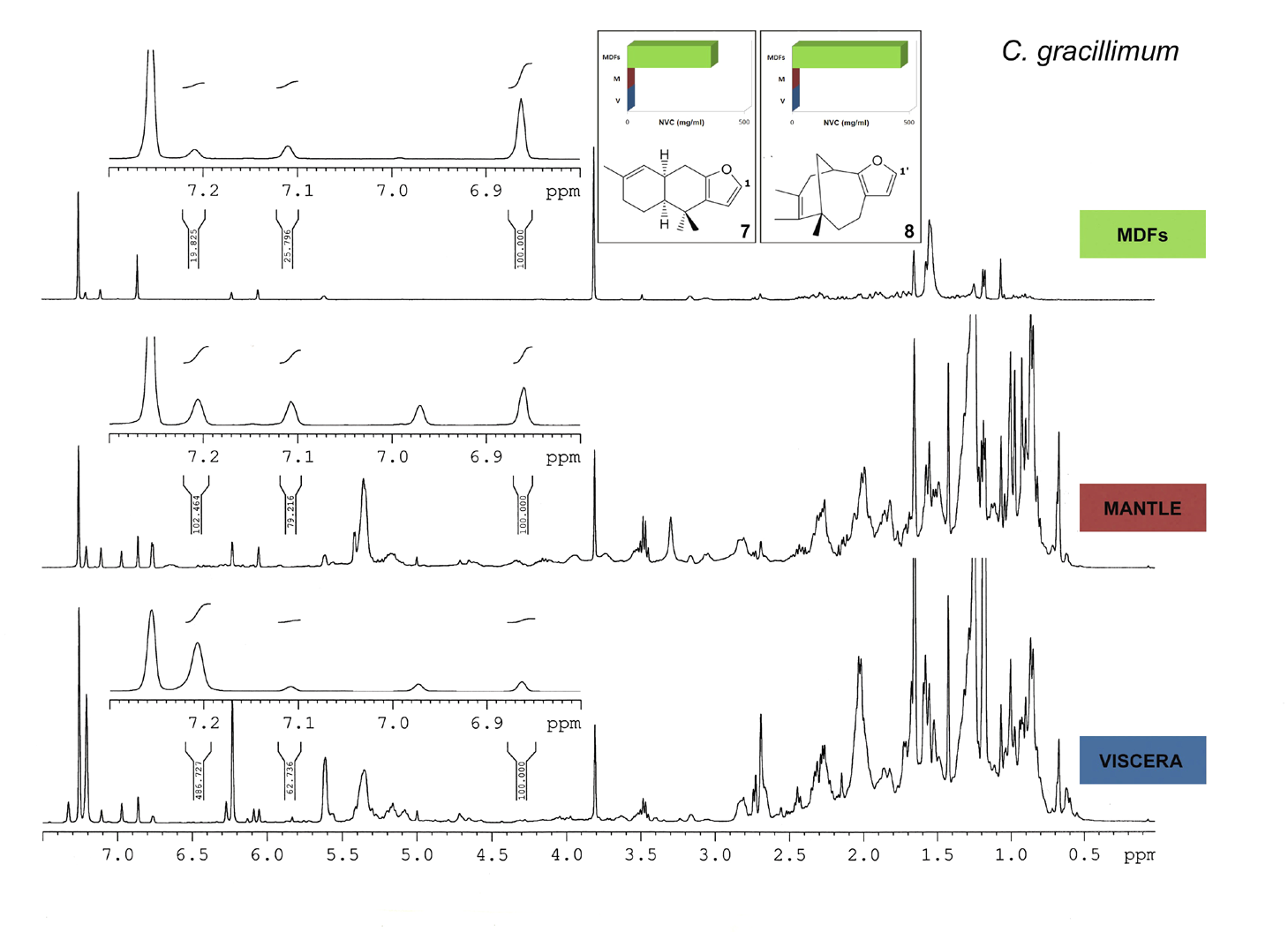

Supplement: Figure S10 — 1H NMR spectra of C. gracillimum. 1H NMR spectra (400 MHz) of crude extracts from one individual of C. gracillimum in CDCl3 containing dimethylfumarate (DMF) as internal standard. Colored bars show the natural volumetric concentration (NVC, mg/ml anatomical section) of compounds 7 and 8 in the different body parts of the nudibranch. (TIF) [file pone.0062075.s010.tif]
